# Supplementary material for: Different Patterns of Platinum Resistance in Ovarian Cancer Cells with Homologous Recombination Proficient and Deficient Background
Source: Int J Mol Sci. 2024 Mar 6;25(5):3049. doi: 10.3390/ijms25053049 (PMC10932017; doi:10.3390/ijms25053049)

Supplementary materials

Supplementary Table S1. Primers used for Real Time-PCR.

GENE	PRIMER FORWARD	PRIMER REVERSE
β-*Actin*	GGCTGTATTCCCCTCCATCG	CCAGTTGGTAACAATGCCATGT
ATP7A	GAGCGTCAGATCCTGCCAG	GTAGAAGTACCAGCCGCCAC
ATP7B	GCTCTTTGTGTTCATCGCCC	GCTTCTGTGGCTTGGAGTGA
CTR1	ACACCTGGAGAAATGGCTGG	TTTCGAAGCAGACCCTCTCG
CTR2	AGGCCGTGCTTCTCTTTGAT	CTTTGCCAACCTTGATGCCC
MDR1	TGTCAGCTGGTATTTGGGCA	CAGTTCTGATGGCTGCTAAGAC
LRRC8D	CTGCCTCTACACTCTCTTCTGGC	CGCAAAGTCGTTCTTGACATC
REV7	GGTGGTGGTGGTGATTTTGG	TCCACATGAGACAGGAGGGA
TP53BP1	TAGCCCGCTATCTGATGTGG	ACAGGGATGTCTTTGCTGGG
SHLD1	CAGGGAAGCAGCAACTCTCT	GTTCTCAGAGTCCCAGGAGC
ERCC1	CTGGGAATTCGGTGAGGTGA	AGTCTGGATGGAGGTTGTGG
XPG	GGGCCCGGCATGTCTATAAG	CCCAGTAGATAGGCCAAGTTGA
XPF	GAAGCTCTACCTGTGGCCAA	CGTCTGAATGGCAAGCATGG
PRKDC	TCACCACAACCCTGCTCATC	GCTCATGGGCTCACAGATCA
LIG IV	ACCACGAGAAGGCAAGGATG	GCAATCATGGCAAAGTCCCC
XRCC6	ATGGCCGTGACAGTGCTAAA	ACGGATACATCAAAGCCTCCTG

Supplementary Figure S1. Resistant sublines characterization. A. Growth curves of F3 (blue line), F3 ddpR (green line), Brca1-/- (pink line) and Brca1 -/- ddpR (orange line) cells at different times from seeding. Data are the mean ± SD of six replicates. **B**. ID8 F3 and Brca1-/- parental and resistant cells. For each cell line a sample image was acquired with 40x magnification.

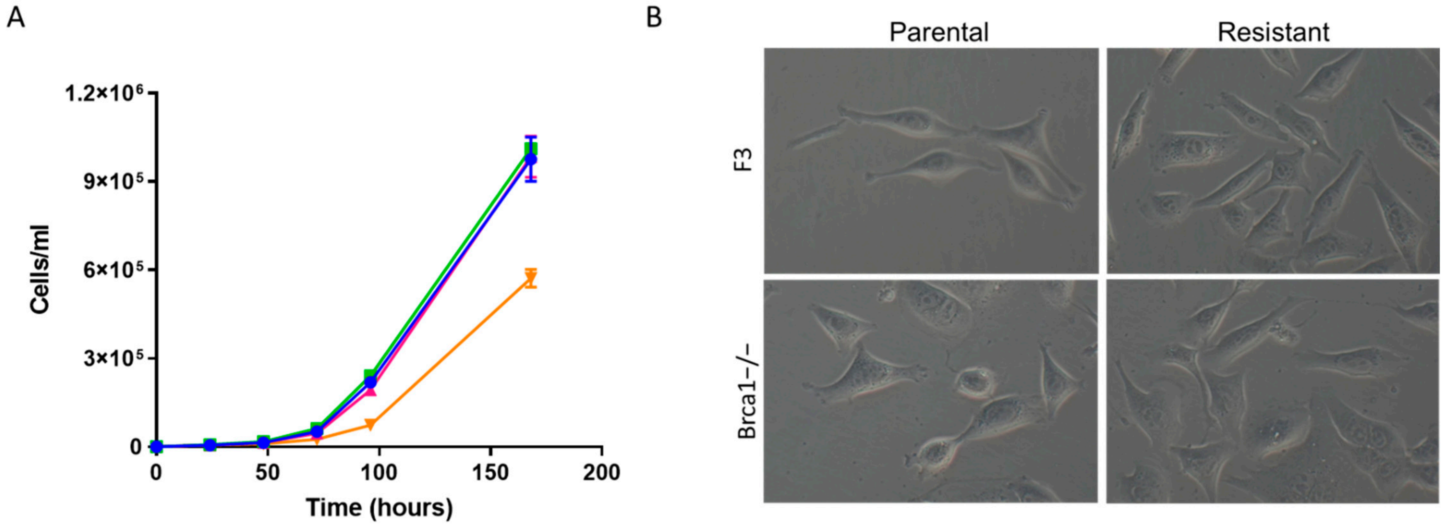

Supplementary Figure S2. Pharmacological characterization of F3, F3 ddpR, Brca1-/- and Brca1-/- ddpR cells. Dose response curves of carboplatin (**A**), oxaliplatin (**B**), paclitaxel (**C**), doxorubicin (**D**), Yondelis (ET-743) (**E**), olaparib (**F**), rucaparib (**G**), niraparib (**H**), ATMi (**I**), ATRi (**J**), Wee1i (**K**),Chk1i (**L**) and DNA PKi (**M**) in F3 (blue line), F3 ddpR (green line), Brca1-/- (pink line) and Brca1 -/- ddpR (orange line) cells. Data are the mean ± standard deviation (SD) of three or four independent experiments.

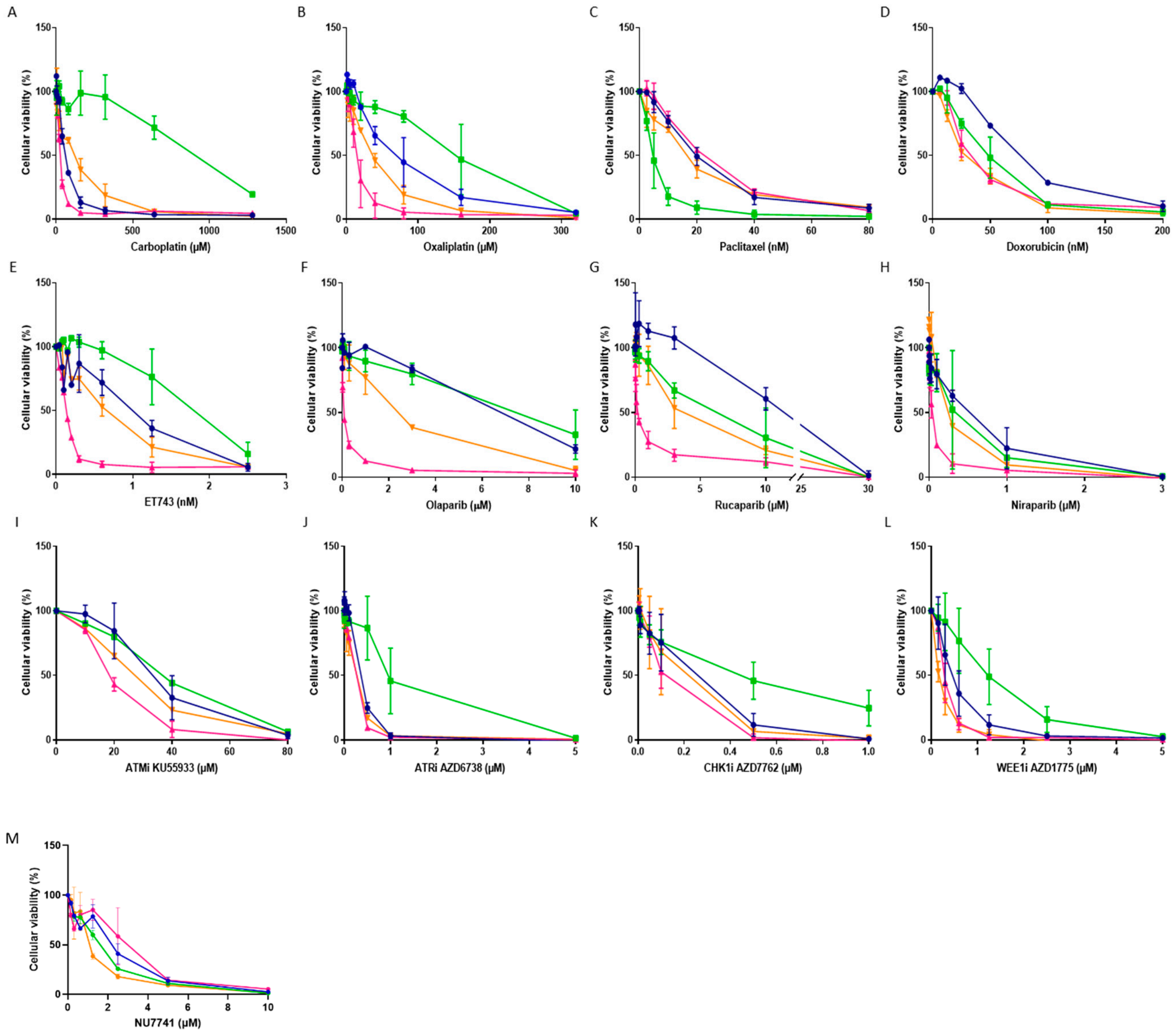

Supplementary Figure S3. Combination of DDR inhibitors and cisplatin. A. Normalized IC_{50} isobolograms showing the synergistic effects of the combination of cisplatin with the inhibitors of ATR, Wee1 and Chk1. **B**. Normalized IC50 isobolograms showing the synergistic effects of the combination of ATRi and WEE1i.

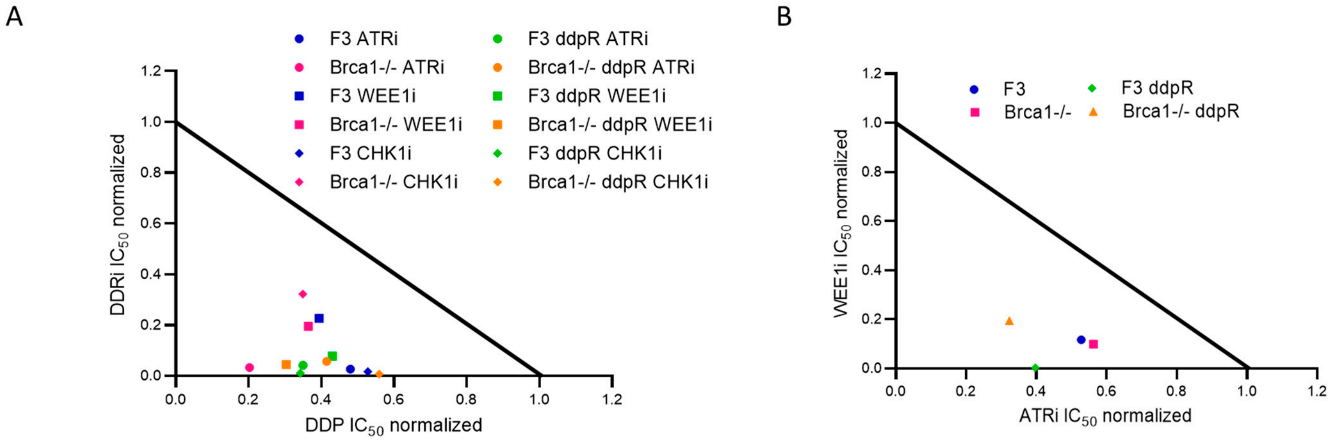

Supplementary Figure S4. DNA repair in sensitive and resistant cells. A. F3, F3 ddpR, Brca1-/- and Brca1 -/- ddpR cells untreated (CTRL) or irradiated with IR 10Gy were stained with DAPI (nuclear staining, blue) and with anti-RAD51 antibody (green). Objective: 40×. **B.** mRNA levels of ERCC1, XPF and XPG in F3 ddpR cells. Values are the mean + SD of two independent experiments, run in triplicate and normalized on the actin gene. Data are expressed as fold-change over the parental cells (dotted line). **C**. mRNA levels of PRKDC, LIG IV and XRCC6 in F3 ddpR cells. Values are the mean + SD of two independent experiments, run in triplicate and normalized on the actin gene. Data are expressed as fold-change over the parental cells (dotted line). **D**. mRNA levels of PRKDC, LIG IV and XRCC6 in Brca1-/- ddpR cells. Values are the mean + SD of two independent experiments, run in triplicate and normalized on the actin gene. Data are expressed as fold-change over the parental cells (dotted line). Only statistically significant differences are reported. For statistical analyses unpaired t-test was used. *=p<0.05; **=p<0.005.

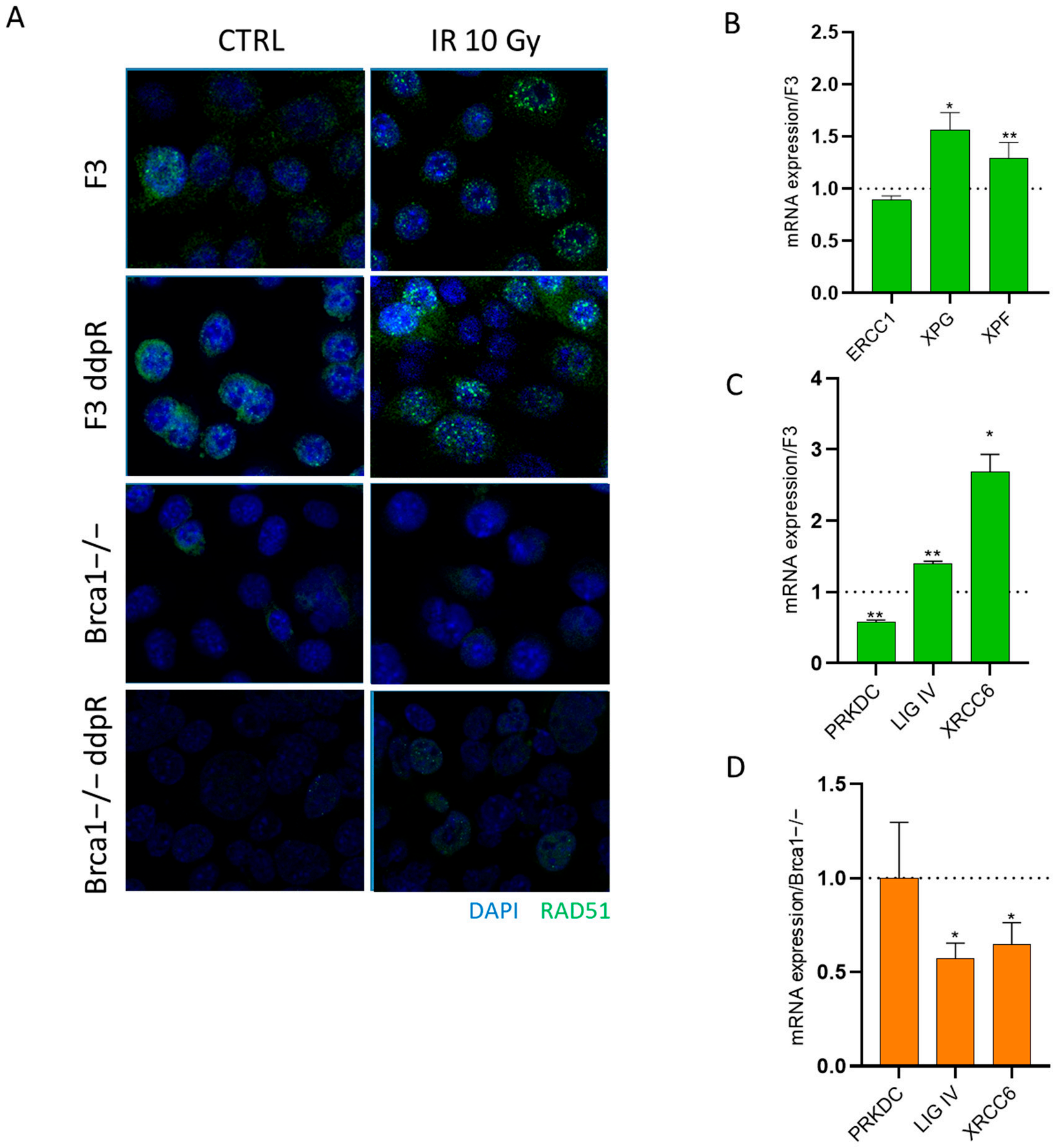

Supplementary Figure S5. Metabolic stress in F3 and Brca1-/- parental and ddpR sublines. A. ROS induction in F3 (blue) and F3 ddpR (green) cells at basal levels and after 24h IC50 dose metformin treatment. Dose response curves of rotenone (**B**) and fenformin (**C**) in F3 (blue line), F3 ddpR (green line), Brca1-/- (pink line) and Brca1 -/- ddpR (orange line) cells. Data are the mean ± standard deviation (SD) of three or four independent experiments. **D.** Transmission electron microscopy of mitochondria in F3 and F3 ddpR cells. Scale bar: 1µm left panels, and 500nm right panels. **E.** Glycolitic ATP-production rate in Brca1-/- and Brca1-/- ddpR cells. **F**. Normalized caspase activity at basal level in Brca1-/- (pink) and Brca1-/- ddpR (orange) cells at 24, 48 and 72h after seeding. **G.** Quantification of annexin V positive cells at basal condition at 24, 48 and 72h after seeding in Brca1-/- (pink) and Brca1-/- ddpR (orange) sublines.

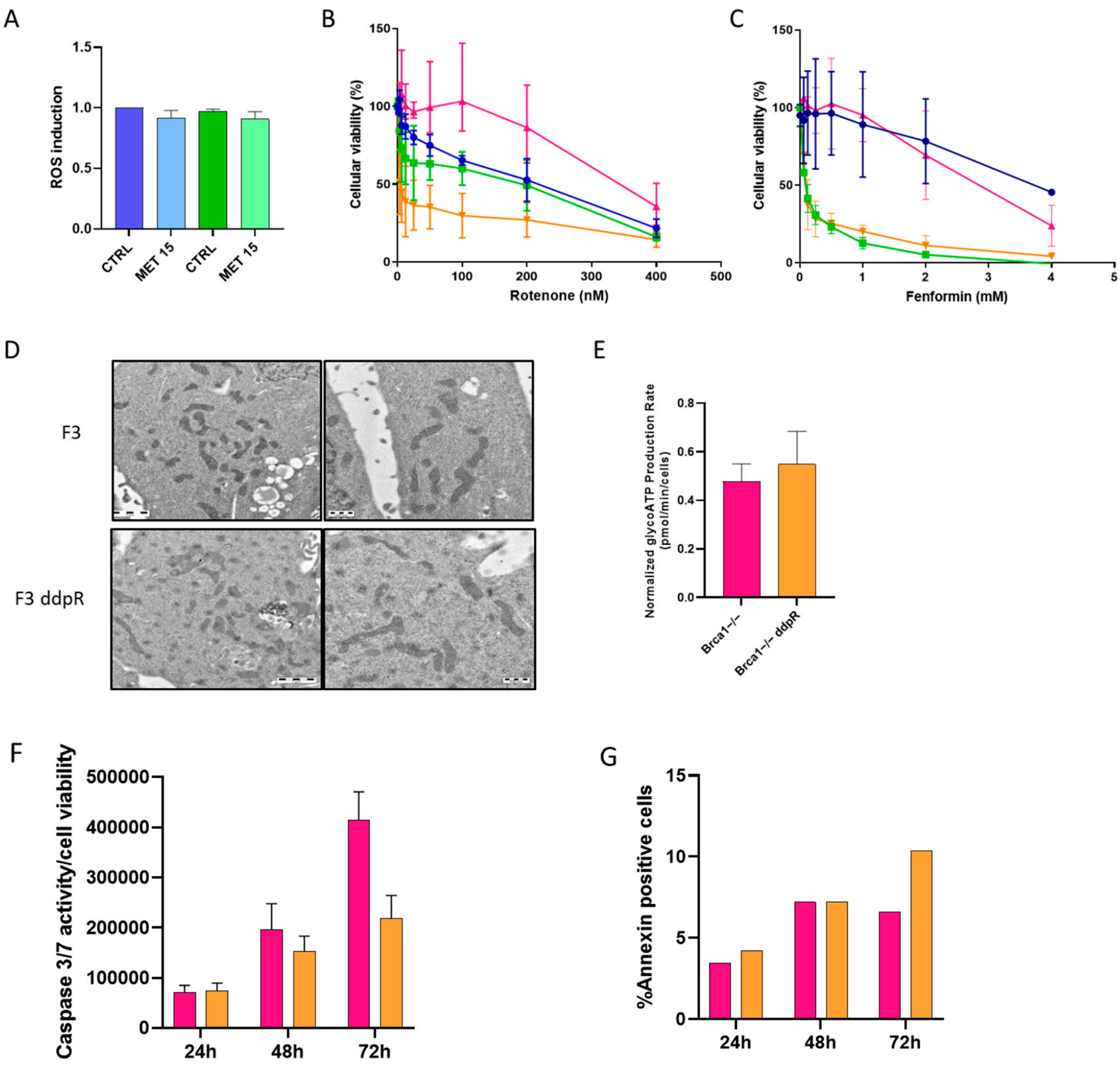

Supplementary Figure S6. Uncropped versions of cropped western blots. Uncropped version of gels showed in Figure 2A.

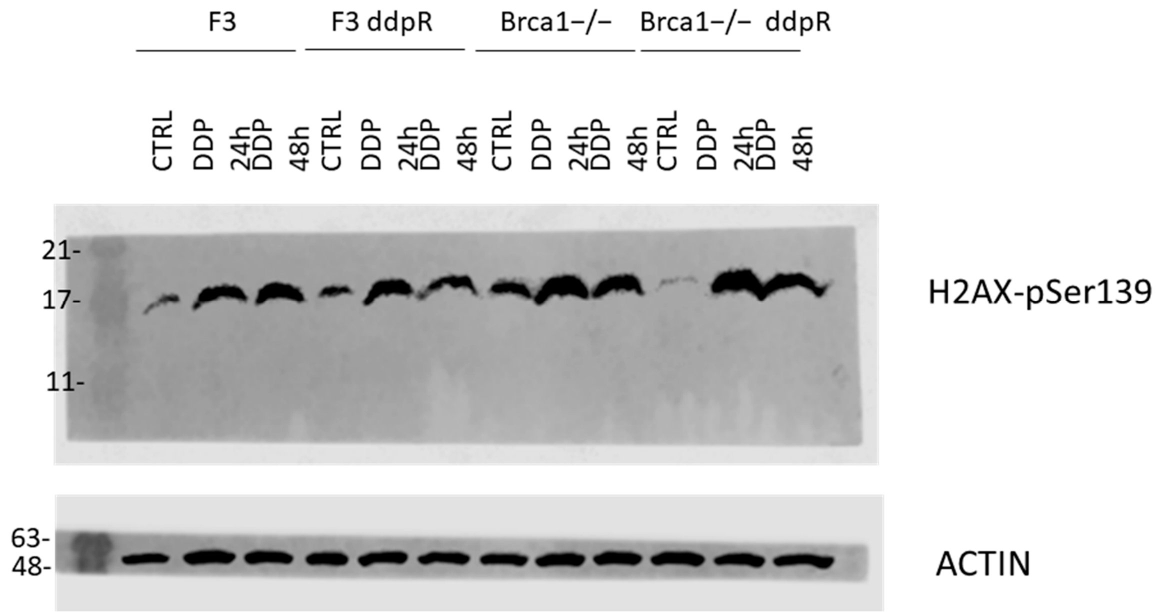

Supplement: Supplementary file 1 [file ijms-25-03049-s001.zip › ijms-2857284-supplementary.pdf]
